# Sometimes they come back: endocytosis provides localization dynamics of a subtilase in cells committed to cell death

**DOI:** 10.1093/jxb/erz014

**Published:** 2019-01-21

**Authors:** Svetlana V Trusova, Sergei A Golyshev, Nina V Chichkova, Andrey B Vartapetian

**Affiliations:** Belozersky Institute of Physico-Chemical Biology, Moscow State University, Moscow, Russia

**Keywords:** Cell death, clathrin-mediated endocytosis, protein localization, subtilisin-like protease, phytaspase

**Proteins are synthesized in the cytoplasm of cells and delivered to their destinations with the aid of targeting signals within their amino acid sequences and through interactions with other proteins. However, a number of proteins are capable of further re-localization in response to external and internal cues, thus allowing the cell to adapt to changing environmental conditions. For plant subtilisin-like proteases (subtilases), secretion of mature enzymes into the extracellular space, the apoplast, has until now been considered to be the end point of their journey. But this may not be the case, and here we present and discuss data showing that for extracellular death-promoting plant subtilases, clathrin-mediated endocytosis provides a gateway to re-enter cells committed to cell death.**

Subtilases comprise the largest family of serine peptidases in plants and possess diverse functions, from ‘trivial’ protein degradation to delicate regulation of vitally important processes, such as growth and development, the immune response, and cell death (Schaller *et al.*, 2018). They are synthesized as precursor proteins containing a N-terminal signal peptide that drives secretion of the vast majority of the mature proteins into the apoplast. It has been known for some time that for phytaspases (plant aspartate-specific proteases), which are subtilase-family members, a further trafficking step may exist (Chichkova *et al.*, 2012). Phytaspases differ from other subtilases in their strict aspartate cleavage specificity, resembling that of caspases, which are animal apoptotic proteases (Galiullina *et al.*, 2015). Also similar to caspases, *Nicotiana tabacum* phytaspase has been shown to be essential for programmed cell death (PCD) induced by various biotic and abiotic cues (Chichkova *et al*., 2004, 2010). Notably, PCD-inducing stresses have been reported to cause rapid phytaspase re-localization from the apoplast to inside the cell, with concomitant and rapid (within 1 h) appearance of phytaspase proteolytic activity within the cell (Chichkova *et al*., 2004, 2010). To date, the mechanisms responsible for phytaspase re-import have remained elusive. As stress-induced PCD in plants frequently causes damage to the plasma membrane (van Doorn *et al.*, 2011), the possibility of plasma membrane permeabilization as a cause of phytaspase internalization may not be completely excluded. However, we have recently obtained data that indicate that a mechanism for active retrograde trafficking of phytaspases exists in plant cells.

## Identifying a pathway for active phytaspase internalization in a model system

Phytaspases from *N. tabacum* (NtPhyt) and some other plants are known to accumulate in the apoplast, as shown during transient production by means of agro-infiltration in *Nicotiana benthamiana* leaves with mRFP or EGFP fluorescent tags visualized by confocal fluorescence microscopy. This localization is consistent with that of endogenous phytaspases, as determined by fractionation experiments and proteolytic activity measurements. It is the PCD induction that causes redistribution of the enzyme towards the cell interior. However, Arabidopsis phytaspase (AtPhyt), which we have identified recently (Chichkova *et al.*, 2018), behaves in a different way. While displaying predominantly apoplastic localization in non-stressed epidermal cells of Arabidopsis leaves, AtPhyt-EGFP demonstrates dynamic localization when transiently produced in *N. benthamiana* epidermal cells (Box 1). During the first 2 d after leaf infiltration, AtPhyt was visualized, as expected, as an apoplastic protein. However, at day 3, accumulation of AtPhyt-EGFP inside the cells in the form of fluorescent dots became evident, with concomitant disappearance of the protein from the apoplast (see Box 1, images A, B). This re-localization occurred spontaneously in the absence of any additional treatment of the sample.

**Box 1. Spontaneous re-localization of AtPhyt from the apoplast to inside *N. benthamiana* cells is prevented by inhibition of clathrin-mediated endocytosis**

Expression of the AtPhyt-EGFP protein in *N. benthamiana* leaves was achieved by agro-infiltration. At 2 d post-infiltration (p.i.), confocal fluorescence microscopy reveals AtPhyt-EGFP to be an apoplastic protein (A), whereas at 3 d p.i. AtPhyt-EGFP forms multiple dots inside the cell (B). Notably, NtPhyt-mRFP remains apoplastic in the absence of PCD-inducing stress at any time point (C–H). Staining of the AtPhyt-EGFP producing leaves with Evans Blue does not reveal cell death (I), in contrast to leaves treated with antimycin A. In the presence of an inhibitor of clathrin-mediated endocytosis (mRFP-Hub1), no evidence for AtPhyt-EGFP re-localization can be observed (J–L). Note that mRFP alone does not prevent internalization of AtPhyt-EGFP (M–O). Expression of the target genes in the plant cells was driven by the constitutive 35S promoter. Data were reproducible over three independent experiments.

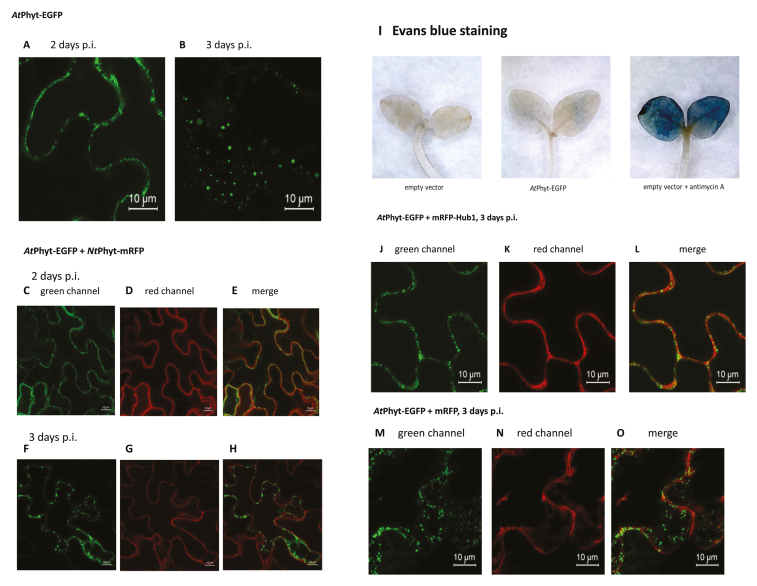

To account for this unanticipated behavior, and because the intracellular localization pattern of AtPhyt in non-stressed cells resembled that of NtPhyt in PCD-induced cells, we initially hypothesized that synthesis of AtPhyt in the *N. benthamiana* system could induce a PCD-like response that triggers phytaspase internalization. To test this, we used NtPhyt-mRFP as a PCD sensor by producing it simultaneously with AtPhyt-EGFP in *N. benthamiana* cells by co-infiltration. By the 3-d time-point and subsequently, when AtPhyt-EGFP had already undergone intracellular localization, no redistribution of NtPhyt-mRFP from the apoplast to inside the cell was evident (Box 1, images F–H), which argued against our hypothesis. Consistently, staining of the AtPhyt-EGFP-producing leaves with Evans Blue failed to reveal cell death (Box 1, image I). Simultaneous staining of the AtPhyt-EGFP-producing cells with FM4-64 dye, an endocytic tracer, allowed visualization of intracellular FM4-64-positive membranous vesicles, and notably some of them co-localized with the AtPhyt-EGFP signal. This observation prompted the idea that AtPhyt could possibly enter plant cells via an endocytic pathway.

In plants, clathrin-mediated endocytosis is a prominent (or at least, the best understood) form of endocytosis (Paez Valencia *et al.*, 2016). Specific inhibition of this pathway can be achieved by over-production of the C-terminal fragment of clathrin heavy chain (known as Hub), which then acts in a dominant negative fashion (Dhonukshe *et al.*, 2007; Kitakura *et al.*, 2011; Li and Pan, 2017). To test the hypothesis that clathrin-mediated endocytosis could be responsible for AtPhyt internalization under normal (non-stressed) conditions, we produced AtPhyt-EGFP protein in *N. benthamiana* leaves either alone or in combination with mRFP-Hub. Three days after infiltration, individually expressed AtPhyt-EGFP displayed a typical pattern of internalization whereas co-expression of mRFP-Hub completely prevented retrograde transport of AtPhyt (Box 1, images J–-L), thus pointing to clathrin-mediated endocytosis as a mechanism for AtPhyt internalization.

## Stress-induced retrograde trafficking of phytaspases relies on clathrin-mediated endocytosis

Is the same endocytic pathway employed by ‘canonical’ phytaspases for internalization in the course of PCD? To address this possibility, NtPhyt-mRFP protein was expressed in *N. benthamiana* leaves either alone or in combination with EGFP-Hub, and localization of NtPhyt was studied both before and after induction of oxidative stress by treatment with antimycin A. As shown in Box 2, as expected, NtPhyt-mRFP expressed alone responded to antimycin A treatment by re-localization from the apoplast to the cell interior, as visualized by multiple small fluorescent dots inside the cell (Box 2, images A, B). Notably, co-expression of EGFP-Hub completely nullified intracellular accumulation of NtPhyt-mRFP, the enzyme being retained at cell boundaries (Box 2, images C–H).

**Box 2. Inhibition of clathrin-mediated endocytosis prevents oxidative stress-induced NtPhyt internalization**

In accordance with previously reported data (Chichkova *et al.*, 2010), NtPhyt-mRFP protein transiently expressed in *N. benthamiana* leaves exhibits apoplastic localization (A) and is re-localized to the cell interior upon oxidative stress induced by treatment with antimycin A (B; 10 μM antimycin A for 8 h). Simultaneous production of an inhibitor of clathrin-mediated endocytosis (Hub) severely interferes with the stress-induced uptake of NtPhyt-mRFP. As Arabidopsis has two genes encoding clathrin heavy chain, Hub1 (C, E, G) and Hub2 (D, F, H) were assessed separately for their effects. Co-production and localization of NtPhyt-mRFP with EGFP-Hub1 or EGFP-Hub2 under non-stressed conditions are shown in (I–K) and (L–N), respectively. Expression of the target genes in the plant cells was driven by the constitutive 35S promoter. At least three independent experiments of each type were performed, with similar results.

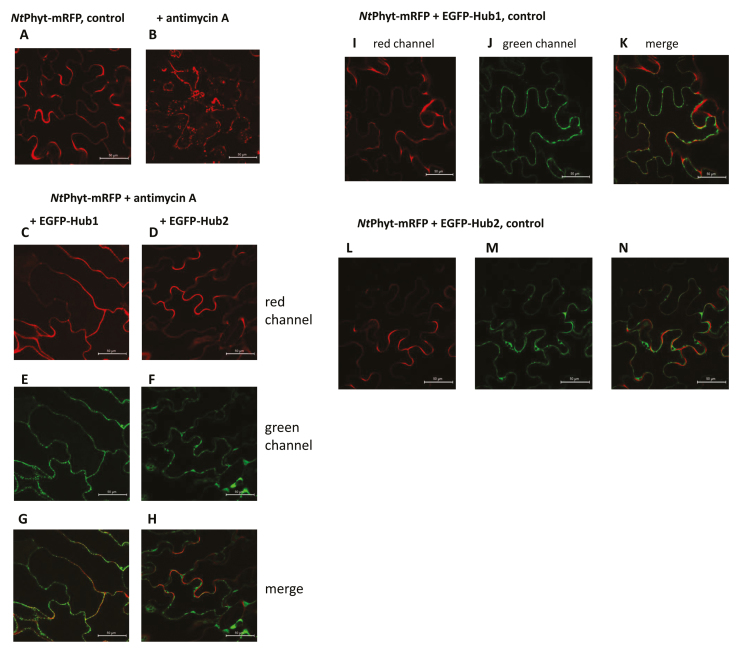

Taken together, these data indicate that cell death-related members of the plant subtilisin-like protease family display dynamic localization (as alluded to in the title of this paper) that is dependent on vesicular membrane trafficking (Box 3). After being secreted into the apoplast, phytaspases can utilize active transport for the delivery of proteolytically active enzyme inside plant cells. Our study points to clathrin-mediated endocytosis as a route for phytaspase entry. While a growing number of plant proteins have been identified that exploit clathrin-mediated endocytosis for their functioning (Geldner and Robatzek, 2008; Reynolds *et al.*, 2018), this type of ‘mobility’ appears to have no precedent among plant proteases.

**Box 3. Schematic representation of vesicular membrane trafficking pathways of phytaspases**

Phytaspases are synthesized as pre-proenzymes equipped with an N-terminal signal peptide for secretion. On their way out of the cell, proenzymes are autocatalytically and constitutively processed, and the mature proteases released into the apoplast (Chichkova *et al.*, 2010). Brefeldin A (BFA), an exocytosis inhibitor, prevents phytaspase externalization. The re-import of phytaspase from the apoplast into *N. benthamiana* cells either requires induction of PCD (for NtPhyt and for a number of phytaspases from other plant species; red arrow) or occurs spontaneously after a 2-d lag period (for AtPhyt; blue arrow). In both cases, retrograde trafficking of phytaspases is nullified by Hub, an inhibitor of clathrin-mediated endocytosis. Q1–Q3 refer to important new questions that are raised by the dynamic localization of phytaspases (see text for discussion). A speculative phytaspase recycling step is shown by the dashed arrow (see Question 2 in the text).

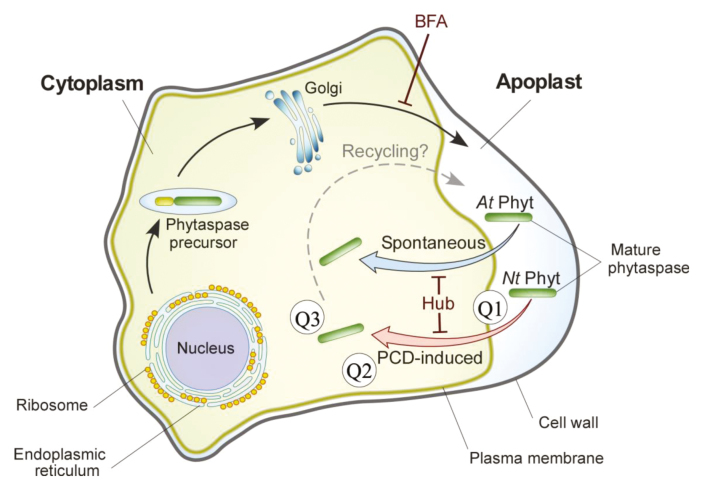

## Emerging questions related to dynamic localization of phytaspases

### Question 1: how can phytaspase become endocytosed?

Clathrin-mediated endocytosis targets proteins that localize at the plasma membrane and possess cytoplasmic domains, to which clathrin is recruited with the aid of adapter proteins (Chen *et al.*, 2011; Traub and Bonifacino, 2013). However, there is currently no evidence for the association of phytaspases with the plasma membrane. Phytaspases do not contain transmembrane domains and are soluble proteins that can easily be obtained in the apoplastic wash.

Thus, it is a reasonable hypothesis that there is a cell surface receptor for phytaspases. This putative receptor would have to be abundant, or extremely efficient, to successfully cope with internalization of an abundant ligand, especially when phytaspases are over-produced (as shown in Boxes 1, 2). It might be phytaspase-specific or also act as a receptor for other molecule(s). Since phytaspases are proteases, they might cleave this putative receptor upon binding, with functional consequences. In animal cells, a family of so-called protease-activated receptors is known that induce intracellular signaling upon cleavage of the extracellular domain of the receptor by cognate extracellular proteases (Soh *et al.*, 2010; Flaumenhaft and De Ceunynck, 2017). However, this does not appear to be a mechanism for protease delivery into the cell.

### Question 2: why is phytaspase internalization normally not constitutive but occurs rapidly upon induction of PCD?

Among possible explanations are PCD-induced modifications of phytaspases or of their putative receptor (or both) to allow the interaction, or the inactivation of a hypothetical repressor that could interfere with phytaspase recognition. An additional possibility is worth mentioning. In some cases, cargo internalized by an endocytic pathway can be recycled back to the plasma membrane (Paez Valencia *et al.*, 2016). It can be imagined that perhaps internalization/recycling is the *modus vivendi* for phytaspases, with recycling prevailing over internalization under normal conditions. In this scenario, an impact from PCD might consist of a ‘simple’ shifting of the equilibrium by suppressing recycling. Two related questions arise: is PCD the sole trigger for NtPhyt internalization? Can some other naturally occurring signals induce NtPhyt uptake?

In the case of the spontaneous AtPhyt uptake by *N. benthamiana* cells that starts after a 2-d lag period, it would be instructive to determine what happens to phytaspase or to the cells during this lag period to allow phytaspase internalization. Is it merely the phytaspase level in the apoplast that matters, or are there other determinants?

### Question 3: is the internalized phytaspase subject to degradation?

A frequent outcome of endocytosis is the delivery of internalized proteins to the vacuole (in plants) or to lysosomes (in animals) with subsequent degradation of the cargo. A related and instructive example is provided by the animal subtilisin-like protease PCSK9. Upon binding of PCSK9 to the complex formed by the low-density lipoprotein receptor and its low-density lipoprotein cholesterol ligand, the ternary complex is stabilized, internalized through a clathrin-mediated endocytic pathway, and transported for lysosomal degradation of all three constituents (Nassoury *et al.*, 2007; Durairaj *et al.*, 2017). However, for phytaspases this destructive scenario seems unlikely for (at least) two reasons. First, the experimentally determined levels of phytaspase proteolytic activity in both healthy tissues and tissues committed to cell death are roughly the same. Second, there is no apparent need to degrade PCD-promoting proteases early in the course of PCD. How then do phytaspases manage to escape from the degradation pathway, and what is the end point of their route inside the cell? Finding answers to these questions is important for understanding the complex mobility of proteolytic enzymes, as well as for understanding the vast array of regulatory approaches that can be utilized by plant cells.
